# The Demographic Representativeness and Health Outcomes of Digital Health Station Users: Longitudinal Study

**DOI:** 10.2196/14977

**Published:** 2020-06-26

**Authors:** Leah Flitcroft, Won Sun Chen, Denny Meyer

**Affiliations:** 1 Faculty of Health, Arts & Design Swinburne University of Technology Hawthorn Australia

**Keywords:** population health, health behavior, health technology, eHealth, health status

## Abstract

**Background:**

Digital health stations offer an affordable and accessible platform for people to monitor their health; however, there is limited information regarding the demographic profile of users and the health benefits of this technology.

**Objective:**

This study aimed to assess the demographic representativeness of health station users, identify the factors associated with repeat utilization of stations, and determine if the health status of repeat users changed between baseline and final health check.

**Methods:**

Data from 180,442 health station users in Australia, including 8441 repeat users, were compared with 2014-2015 Australian National Health Survey (NHS) participants on key demographic and health characteristics. Binary logistic regression analyses were used to compare demographic and health characteristics of repeat and one-time users. Baseline and final health checks of repeat users were compared using McNemar tests and Wilcoxon signed rank tests. The relationship between the number of checks and final health scores was investigated using generalized linear models.

**Results:**

The demographic profile of SiSU health station users differs from that of the general population. A larger proportion of SiSU users were female (100,814/180,442, 55.87% vs 7807/15,393, 50.72%), younger (86,387/180,442, 47.88% vs 5309/15,393, 34.49% aged less than 35 years), and socioeconomically advantaged (64,388/180,442, 35.68% vs 3117/15,393, 20.25%). Compared with NHS participants, a smaller proportion of SiSU health station users were overweight or obese, were smokers, had high blood pressure (BP), or had diabetes. When data were weighted for demographic differences, only rates of high BP were found to be lower for SiSU users compared with the NHS participants (odds ratio [OR] 1.26; *P*<.001). Repeat users were more likely to be female (OR 1.37; *P*<.001), younger (OR 0.99; *P*<.001), and from high socioeconomic status areas—those residing in socioeconomic index for areas quintiles 4 and 5 were significantly more likely to be repeat users compared with those residing in quintile 1 (OR 1.243; *P*<.001 and OR 1.151; *P*<.001, respectively). Repeat users were more likely to have a higher BMI (OR 1.02; *P*<.001), high BP (OR 1.15; *P*<.001), and less likely to be smokers (OR 0.77; *P*<.001). Significant improvements in health status were observed for repeat users. Mean BMI decreased by 0.97 kg/m2 from baseline to final check (z=−14.24; *P*<.001), whereas the proportion of people with high BP decreased from 15.77% (1080/6848) to 12.90% (885/6860; χ^2^_1_=38.2; *P*<.001). The proportion of smokers decreased from 11.91% (1005/8438) to 10.13% (853/8421; χ^2^_1_=48.4; *P*<.001). Number of repeat health checks was significantly associated with smoking status (OR 0.96; *P*<.048) but not with higher BP (*P*=.14) or BMI (*P*=.23).

**Conclusions:**

These findings provide valuable insight into the benefits of health stations for self-monitoring and partially support previous research regarding the effect of demographics and health status on self-management of health.

## Introduction

### Background

Technological advances in recent years have changed the way consumers access health care and enabled the use of a range of digital mechanisms for self-monitoring of health, including mobile phone apps, wearable trackers, and web-based monitoring systems [1,2]. Although self-monitoring in general has been shown to be effective in the management of health risk factors and chronic disease and in increasing self-efficacy in disease management [3-5], limited research has been conducted to date regarding the long-term benefits of using digital technologies to self-monitor health [6,7]. Some benefits have been identified in terms of supporting behavior change [7,8], promoting weight loss [7,9,10], increasing physical activity [7,8,10,11], assisting with smoking cessation [7,10], and improving self-management of chronic disease [7,12], particularly in those with more serious chronic illness [6]; however, findings to date have been inconsistent.

The increased utility of digital self-monitoring technology over traditional paper-based tracking, in terms of it enabling the easy collection and exchange of health-related information between consumers and health care providers, has also been supported by a number of authors [13-15]. There is some evidence that data collected through such technologies is more reliable than data collected via other means such as manual measurement, self-report, and in some cases medical professionals [16-18]. The mechanisms behind these findings are varied and include the competence of the general public in using and interpreting medical instrumentation such as sphygmomanometers [19], social desirability associated with self-reports of disease and risk factors [16,20], and a phenomenon known as *white coat syndrome*, in which blood pressure (BP) and heart rate are artificially elevated in a clinical setting [21,22].

The uptake and utilization of health monitoring devices and apps appear to be influenced by a range of social, health-related, and demographic factors, including age [23-25], health status [25-27], and socioeconomic status (SES) [23,25,28]. However, it is possible that the influences of such characteristics may be dependent on the type of technology in question [23].

Given the potential benefits to both consumers and health professionals, and the rapid increase in the use of technology in the health arena, there is some concern surrounding the influence of sociodemographic characteristics on access to digital technologies [23,29]. Such influences may result in the potential for certain disadvantaged subgroups to be *left behind* in the digital health age and unable to benefit from the potential of such technologies [23]. Older age and socioeconomic disadvantage, in particular, are often associated with barriers to the utilization of digital health apps, due to poor levels of electronic health literacy, prohibitive costs, and limited access to the internet [23,30].

The SiSU Health Group is a health and wellness company that aims to ease the effects of lifestyle-related diseases on global health care systems through the use of technology. Their health check stations, installed in a number of locations across Australia, offer an affordable and accessible platform to help people live a healthier life. SiSU health stations are free of charge for all Australians aged 16 years and older, providing an alternative method of enabling the general population to monitor their health and access relevant health information. These stations have the potential to reach consumers who face barriers to the utilization of digital health technologies [29-31], particularly those facing economic barriers to such technologies. Although several studies have been published that investigate the determinants of health kiosk utilization, findings are varied. Socioeconomic factors including income, employment status, country of birth, gender, and age have all been found to influence the utilization of health kiosks to varying degrees [29-33]; however, the results are inconsistent.

There is some evidence to demonstrate that the utilization of health kiosks to access health information can lead to increased screening rates, improved health literacy, and a reduction in the burden on medical services [33]. The majority of these studies, however, investigated the utilization of kiosks that provide health information only, with very few including kiosks that enable health measurement and self-monitoring [29-32] such as the SiSU health station. Limited information is available regarding the potential benefits of health kiosk utilization for self-monitoring in terms of improved health status and a reduction in health risk behaviors. For this reason, this study aimed to provide insight into the demographic and health-related characteristics that are associated with the utilization of health kiosks for self-monitoring and identify any observed improvements in the health status of users over time, using data obtained from SiSU health stations. These stations are installed in a number of pharmacies, retail outlets, and workplaces throughout Australia and the United Kingdom. In Australia, the majority are currently located in Priceline Pharmacies.

### Objectives

The SiSU wellness health check station collects data on a range of self-reported and machine-measured health indicators, including diabetes status, physical activity levels, waist circumference, dietary practices, heart rate, BP, weight, BMI, and body fat percentage. The SiSU station is designed to be a vertical space with mobile 3G connection or access to a private Wi-Fi network. Users engage with the station for approximately 7 min to answer a series of questions and to provide various health-related measures, before they receive immediate feedback about their health status on the screen of the station. Users of the health stations are able to monitor their progress and health changes over time by connecting their health check station profile to a free app developed by SiSU Health Group and downloadable from Google Play or the iTunes store. The stations and associated apps are intended to assist consumers by allowing them to monitor their health status over time and providing alerts to consumers when follow-up with a general practitioner is recommended [34].

There is a lack of evidence regarding the health benefits of kiosks that allow self-monitoring of health outcomes and inconsistent evidence regarding the demographic of users. This study, therefore, aimed to determine if the users of SiSU health stations in Australia differ from the general population in terms of demographics and health status by comparing SiSU health station users with participants of the 2015 National Health Survey (NHS); investigate the demographic and health-related characteristics that are associated with repeated utilization of the SiSU health stations in Australia; and identify if the health status of repeat users of SiSU health stations in Australia improved from baseline (at their first check) to their final health check.

## Methods

### Study Sample

This study uses data collected from 192 SiSU health check stations installed across Australia, for the period October 28, 2017, to June 27, 2018. Due to resource limitations, data for health check stations in the United Kingdom were not considered in this study. This resulted in a total of 271,151 records pertaining to males and females aged 16 years and above in Australia. Users reporting a pregnancy at one or more of their health station checks were removed from the dataset (n=3315). The majority (266,813/271,151, 98.40%) of data were obtained from health check stations installed in Priceline Pharmacies across Australia.

For research question 1—analysis of the demographics and health status of SiSU users—and 2—investigate the demographic and health-related characteristics that are associated with repeated utilization of the SiSU health stations in Australia—only the first health checks of users were included to avoid bias in measurements introduced through potential improvements in health status as a result of self-monitoring. Therefore, records that were identified as repeat checks were excluded from the analysis, as were any users who had undertaken their first health check in the time before the study period. Invalid measurements were also identified and removed, resulting in a sample size of 180,442 records.

The demographics and health status of the users were compared with those of the participants in the 2014-2015 Australian NHS, a nationally representative survey of 19,000 people in approximately 15,000 households. NHS data are weighted to reflect sampling fractions for each respondent, ensuring that the results are representative of the general population [35]. Only NHS participants aged 16 years and above were included in this analysis, resulting in a total of 15,393 records.

For research question 3—analysis of changes in health status from baseline—only users who had undertaken 2 or more health checks were included. Users were classified as repeat users if their unique user ID appeared more than once in the dataset. Suspected shared accounts were also identified by comparing the age and gender recorded at each health check. User IDs with inconsistent entries for these variables were considered to be shared accounts and were removed from the analysis, resulting in a total of 27,522 health checks pertaining to 8441 users.

### Measures

Data on the following variables were used in this study: gender, age, SES, state, BP, BMI, diabetes status, smoking status, and repeat user status.

The health station questions regarding fruit and vegetable consumption and physical activity are only asked of nondiabetic health station users, resulting in a large amount of missing data for each of these variables (64,959/180,442, 36.00% missing). Therefore, these variables were excluded from this analysis.

The format of the age variable in the NHS data, which was grouped into 5 year categories, necessitated the grouping of the SiSU health station age variable into categories for the purposes of research question 1. To reduce the number of categories, 10 year age groups were selected. For the remaining research questions, age was treated as a continuous variable.

SES was defined using the 2016 version of the index of relative advantage and disadvantage (IRSAD) under the Australian Bureau of Statistics’(ABS) socioeconomic indexes for areas (SEIFA), which summarizes a range of variables that are considered to represent relative socioeconomic advantage and disadvantage. The IRSAD ranks geographical areas on a continuum from most disadvantaged to least disadvantaged based on this summary. Areas are ranked into quintiles, where quintile 1 contains the lowest 20% of areas (most disadvantaged), quintile 2 contains the next lowest 20% of areas, and so forth, resulting in 5 equal-sized groups. SEIFA scores were allocated to SiSU health station users by matching their residential postcode to the SEIFA index. State was recorded as the state in which the person resided at the time of their health check.

BP was grouped into categories based on measured systolic and diastolic readings: a reading of ≥140/90 mm Hg was categorized as high. Similarly, BMI was calculated using a person’s measured height (m) and weight (kg), and values of ≥30.0 kg/m^2^ were categorized as overweight or obese. Binary categories were chosen for these variables to enable a direct comparison of the proportion of participants with or without the respective health conditions (prevalence rates).

Diabetes status and smoking status were recorded as *yes* if a person had an affirmative response to the questions: “Do you have a current diagnosis of type 1 or type 2 diabetes?” and “Are you a current smoker?,” respectively.

### Missing Data

Analysis of missing values was performed on the SiSU wellness data to determine the volume of missing data and identify factors associated with missingness. The total proportion of missing values in the dataset was 5.40% (9744/180,442), with BMI and BP having the highest proportion of missing values at 19.00% (34,284/180,442) and 18.79% (33,923/180,442), respectively. Due to the high rate of missing data, inverse probability weighting (IPW) was used to weight records according to their probability of being a complete record, according to the methodology detailed in the study by Seaman and White [36]. A binary logistic regression was conducted to calculate the IPW, with the variables gender, age, state of residence, and SES included in the model. Separate weights were calculated for each missing variable. All statistical analyses were conducted with these weights applied, using only records with complete data for the variables in each model.

Missing data for physical measurements was higher in the NHS compared with the SiSU wellness data. In the 2014-2015 NHS, physical measurements were taken for height, weight, and BP. A total of 24.29% (3740/15,393) of respondents did not have their BP measured, whereas 26.80% (4125/15,393) did not have their height, weight, or both measured. The NHS utilized imputation to estimate physical measurements for these participants.

### Statistical Analysis

To assess if the users of SiSU health stations are representative of the general population, selected demographic and health characteristics of the sample were compared with the those of the Australian population using data from the 2014-2015 NHS.

Characteristics that were directly comparable between the SiSU health station users and the NHS dataset were age group, sex, SES, state of residence, BP, BMI, diabetes status, and smoking status. Demographic characteristics of the 2 groups were compared, and SiSU health station data were then weighted to account for demographic differences between the SiSU health station users and NHS participants. Using these weighted data, logistic regression was used to obtain odds ratios (ORs) to determine the magnitude of any differences between the health-related measures for SiSU health station users compared with NHS participants. In total, 4 models were created, 1 for each of the health characteristic variables—BMI, BP, diabetes status, and smoking status—with the health characteristic as the dependent variable and *group* (SiSU user or NHS participant) as the independent variable. The demographic variables age, gender, SES, and state were included as controls in these models.

Comparisons were also made between repeat and nonrepeat users of the SiSU health stations. Users were classified as repeat if they had undertaken 2 or more health checks within the time period, whereas users were considered nonrepeat if they had only undertaken 1 health check during the period or had no recorded user ID number. Binary logistic regression was performed to identify the demographic and health-related factors that predict the probability of being a repeat user. With repeat status as the dependent variable, demographic and health predictors included in the model were age, gender, SES, state, BMI, BP category, diabetes status, and smoking status. Binary logistic regression was chosen because of the binary nature of the dependent variable and the ability to introduce covariates for analysis and quantify the relationship between the dependent and independent variables in terms of ORs [37,38].

To establish if the health status of repeat users changed between their first and final health checks, the baseline (first health check) and final (last check identified in the period under study) health measurements of repeat users were compared. McNemar tests were performed for the categorical variables BP category and smoking status to identify any change in proportions between baseline and final checks, and a Wilcoxon signed rank test was performed for baseline and final BMI measurements to identify any change in mean BMI scores from baseline to final check. Both methods were chosen because of their ability to allow the comparison of related or paired samples, and both the methods are often used in research comparing pre and posttreatment measurements [39,40].

Finally, to determine if the number of health checks completed by a user affects health outcomes at their final check, binary logistic regression models were constructed for each of the health outcomes BP category and smoking status, again because of their ability to include covariates and produce ORs for the quantification of relationships. A generalized linear model was used for BMI, assuming a gamma distribution due to the skewness of the data. This is a method suggested by some authors to overcome the issue of right-skewed data while avoiding issues associated with log-retransformation [41]. Baseline measurements were included in these models to control for differences in baseline health scores, and the demographic variables gender, age, and SES were included as covariates.

A *P* value of less than .05 (2-tailed) was deemed to be statistically significant. All analyses were performed using International Business Machines SPSS version 21.

## Results

### Comparison of Demographic and Health Characteristics of SiSU Health Station Users and National Health Survey Participants

The demographic characteristics of SiSU health station users compared with NHS users are presented in Table 1, revealing large differences in proportions across all demographic variables under consideration. SiSU health stations users were found to be younger (86,387/180,442, 47.87% vs 5309/15,393, 34.49% aged less than 35 years; *P*<.001) and living in higher SES areas (64,388/180,442, 35.68% vs 3117/15,393, 20.25% in quintile 5; *P*<.001) compared with NHS participants. A higher proportion of SiSU health station users were female (100,814/180,442, 55.87% vs 7807/15,393, 50.72%; *P*<.001), and the proportion of SiSU users living in each state varied from that of NHS participants, with a larger proportion of SiSU health station users residing in New South Wales compared with the NHS participants (92,636/180,442, 51.34% vs 4982/15,393, 32.37%; *P*<.001).

Although detailed data are not available for Priceline Pharmacy customers, according to Priceline Pharmacies, 97.00% of their customer base is female [42]. This gender distribution varies greatly compared with that of NHS participants and SiSU health station users.

**Table 1:** Comparison of the demographic characteristics of SiSU health station users and National Health Survey participants.

Variable		SiSU health station users (n=180,442), n (%)	NHS^a^ participants (n=15,393), n (%)	*P* value
**Age group (years)**				<.001
	>24	42,027 (23.29)	2499 (16.23)	
	25-34	44,360 (24.58)	2810 (18.26)	
	35-44	26,905 (14.91)	2623 (17.04)	
	45-54	23,529 (13.04)	2530 (16.44)	
	55-64	22,878 (12.68)	2211 (14.36)	
	65-74	15,101 (8.37)	1604 (10.42)	
	≥75	6452 (3.58)	1117 (7.26)	
**Gender**				<.001
	Male	79,628 (44.13)	7586 (49.28)	
	Female	100,814 (55.87)	7807 (50.72)	
**SEIFA^b^ quintile**				<.001
	1	26,439 (14.65)	2961 (19.24)	
	2	27,899 (15.46)	3019 (19.61)	
	3	25,197 (13.96)	3123 (20.29)	
	4	36,519 (20.24)	3172 (20.61)	
	5	64,388 (35.68)	3117 (20.25)	
**State**				<.001
	New South Wales	92,636 (51.34)	4982 (32.37)	
	Victoria	23,377 (12.96)	3914 (25.43)	
	Queensland	35,513 (19.68)	3039 (19.74)	
	South Australia	9760 (5.41)	1113 (7.23)	
	Western Australia	12,824 (7.11)	1637 (10.63)	
	Tasmania	3240 (1.80)	338 (2.20)	
	Northern Territory	154 (0.10)	115 (0.74)	
	Australian Capital Territory	2938 (1.63)	254 (1.65)	

^a^NHS: National Health Survey.

^b^SEIFA: socioeconomic indexes for areas.

**Figure 1:**
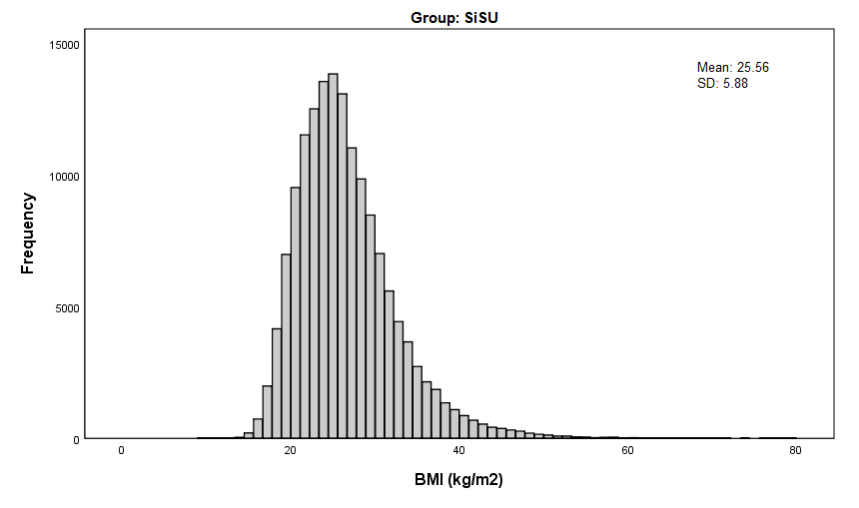
BMI distribution (kg/m2) of SiSU Health Station users, unweighted.

**Figure 2:**
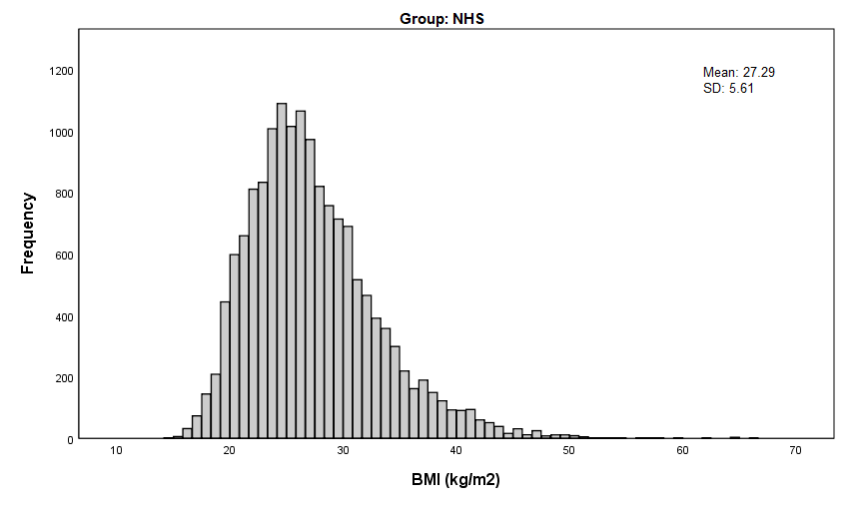
BMI distribution (kg/m2) of National Health Survey participants.

Table 2 provides a comparison of the unweighted and weighted health characteristics of SiSU health station users and NHS participants. Before weighting, SiSU health station users were generally healthier than NHS participants on the variables measured. There was a lower proportion of people with high BP (22,556/140,100, 16.10% compared with 3386/14,690, 23.05%), a lower prevalence of diabetes (9339/180,290, 5.18% compared with 11,909/180,988, 6.58%), and a smaller proportion of people who smoked (22,470/180,481, 12.45% compared with 2149/15,394, 13.96%) in the SiSU health station group. There was also a lower proportion of people with BMI in the overweight to obese range (83,055/149,676, 55.49% compared with 92,651/149,727, 61.88%), with the average BMI almost 2 kg/m^2^ lower for SiSU health station users (25.56 kg/m^2^, SD 5.88 kg/m^2^) compared with NHS participants (27.29 kg/m^2^, SD 5.61 kg/m^2^). Interestingly, however, Figures 1 and 2 demonstrate that the shape of the distribution of BMI for SiSU health station users and NHS participants is almost identical, with both distributions demonstrating right skewness and similar variability.

**Table 2:** Comparison of the health characteristics of SiSU health station users and National Health Survey participants.

Variables		SiSU health station users (unweighted), n (%)	SiSU health station users (weighted), n (%)	NHS^a^ participants, n (%)	NHS participants vs weighted SiSU health station users, OR^b^ (95% CI)^c^	*P* value^d^
**High BP^e^(mm Hg)**						
	Yes	22,556 (16.10)	26,905 (19.16)	3386 (23.05)	1.26 (1.21-1.31)	<.001
	No	117,576 (83.90)	113,227 (80.84)	11,302 (76.95)	N/A^f^	N/A
**BMI (kg/m^2^) status**						
	Overweight/obese	83,055 (55.49)	92,651 (61.88)	9455 (62.07)	1.01 (0.97-1.04)	.66
	Low to normal	66,624 (44.51)	67,245 (38.12)	5,779 (37.93)	N/A	N/A
**Diabetes status**						
	Diabetic	9339 (5.18)	11,909 (6.58)	969 (6.30)	0.95 (0.89-1.02)	.17
	Nondiabetic	171,103 (94.82)	168,533 (93.42)	14,424 (93.70)	N/A	N/A
**Smoking status**						
	Nonsmoker	157,972 (87.55)	156,443 (86.70)	13,244 (86.04)	1.06 (1.01-1.11)	.02
	Smoker	22,470 (12.45)	23,999 (13.30)	2149 (13.96)	N/A	N/A

^a^NHS: National Health Survey.

^b^OR: odds ratio.

^c^Relative odds of exposure in weighted SiSU users compared with NHS participants.

^d^*P* value was obtained from binary logistic regression.

^e^BP: blood pressure.

^f^N/A: not applicable.

Given the demonstrated difference in demographics between the 2 groups, weights were applied to the SiSU health station data to account for these disparities, using the demographic variables age, gender, SES, and state to calculate the weights. The comparison of health characteristics was repeated using these weighted data to determine if SiSU health station users are healthier than the general population when these demographic differences are taken into consideration. ORs were calculated for each health characteristic to assess the magnitude of the difference between the weighted health characteristics of SiSU participants and those of the NHS participants.

Following the weighting of the SiSU health station data, the high BP and smoking variable distributions both demonstrated a significant difference with NHS participants. The proportion of SiSU health station users with high BP remained smaller compared with that of NHS participants (26,905/140,423, 19.16% compared with 3386/14,690, 23.05%), with NHS participants 1.26 times more likely to have high BP compared with SiSU users (*P*<.001). With regard to smoking status, the weighting of the SiSU data did result in a slight increase in the proportion of smokers; however, a smaller proportion of smokers remained in the SiSU group (23,999/180,444, 13.30% compared with 2149/15,394, 13.96%). Although this difference in the proportions was significant, the OR for this relationship was close to 1 (OR 1.06; *P*=.02), indicating that the difference is negligible.

Comparison of the BMI and diabetes status of SiSU health station users and NHS participants demonstrated no significant difference between proportions or an OR close to 1. The difference in the proportion of people with BMI in the overweight to obese range was not significant (92,651/149,727, 61.88% compared with 9455/15,233, 62.07%; OR 1.01; *P*=.66); although there was now a larger proportion of SiSU users with diabetes compared with the NHS participants (11,909/180,988, 6.58% compared with 969/15,381, 6.30%), this relationship was not significant (OR 0.95; *P*=.17).

### Investigation of the Factors That Predict the Repeat Utilization of SiSU Wellness Health Check Stations

Binary logistic regression was used to identify demographic and health-related factors that are associated with repeat utilization of the SiSU health stations. Suspected shared accounts were removed from the analysis, resulting in a sample size of 179,467. Repeat users comprised 4.68% (8441/179,467) of all SiSU health station users in the sample, accounting for a total of 27,522 health checks, with a mean of 3.26 (SD 3.44) checks per person. The average time between baseline and final check for the users in this cohort was 77.60 days, with a minimum of 1 day and a maximum of 283 days between baseline and final check. Overall, 50.84% of users undertook their final check less than 90 days after their baseline check.

Overall, the model was found to be statistically significant (χ^2^_17_=483.5; *P*<.001), but only accounted for 1.00% of the total variance in the dependent variable, repeat user status (Nagelkerke R Square=0.011). Age was found to be significantly associated with being a repeat user, with the odds of being a repeat user decreasing with age; however, this effect size was small (OR 0.992; *P*<.001). Females were 1.371 times more likely to be repeat users (OR 1.371; *P*<.001), whereas those who resided in SEIFA quintiles 4 and 5 were significantly more likely to be repeat users compared with those residing in quintile 1 (OR 1.243; *P*<.001 and OR 1.151; *P*<.001, respectively). Only those residing in the Australian Capital Territory, Victoria, and South Australia had significantly lower odds of being repeat users compared with those residing in New South Wales (Table 3).

In terms of health characteristics, BMI, high BP, and smoking status were all significantly associated with being a repeat user. As BMI increased, the odds of being a repeat user increased; however, the effect size was small (OR 1.020; *P*<.001). Those with high BP were significantly more likely to be repeat users (OR 1.151; *P*<.001), whereas smokers were significantly less likely to be repeat users (OR 0.773; *P*<.001). There was no significant association between diabetes status and being a repeat user (Table 3).

**Table 3:** Binomial logistic regression analysis with the dependent variable: repeat user status.

Independent variable		OR^a^ (95% CI)	*P* value^b^
Age (years)		0.992 (0.991-0.994)	<.001
**Gender (reference=male)**			
	Female	1.371 (1.305-1.441)	<.001
**SEIFA^c^ (reference=quintile 1)**			
	Quintile 2	0.932 (0.837-1.037)	.12
	Quintile 3	1.087 (0.993-1.190)	.07
	Quintile 4	1.243 (1.146-1.349)	<.001
	Quintile 5	1.151 (1.070-1.239)	<.001
**State (reference=NSW^d^)**			
	Australian Capital Territory	0.696 (0.564-0.859)	.01
	Victoria	0.926 (0.859-0.999)	.048
	Queensland	0.968 (0.907-1.032)	.32
	South Australia	0.844 (0.751-0.949)	.01
	Western Australia	0.980 (0.892-1.076)	.67
	Tasmania	0.992 (0.814-1.209)	.94
	Northern Territory	0.609 (0.248-1.494)	.28
BMI (kg/m^2^)		1.020 (1.015-1.024)	<.001
**High BP^e^ (mm Hg; reference=no)**			
	Yes	1.151 (1.070-1.239)	<.001
**Smoking status (reference=nonsmoker)**			
	Smoker	0.773 (0.718-0.831)	<.001
**Diabetes status (reference=diabetic)**			
	Diabetic	0.927 (0.823-1.045)	.22

^a^OR: odds ratio.

^b^*P* value was obtained from binomial logistic regression.

^c^SEIFA: socioeconomic indexes for areas.

^d^NSW: New South Wales.

^e^BP: blood pressure.

### Identify If the Health Status of Repeat Users of SiSU Health Stations in Australia Improved From Baseline to Final Check

A Wilcoxon signed rank test and McNemar tests were performed on the baseline and final health scores for the continuous and binary health characteristic variables, respectively. At baseline, the mean BMI was 26.37 kg/m^2^ (SD 7.43 kg/m^2^), decreasing to 25.40 kg/m^2^ (SD 8.06 kg/m^2^) at the final check. Results of the Wilcoxon signed rank test indicated that this change in mean BMI scores was significant (Z=−14.24; *P*<.001; Table 4).

The proportion of people with high BP decreased from baseline (5768/6848, 15.77%) to final check (885/6850, 12.92%). The results of the McNemar test confirmed that this change in proportions was significant (χ²_1_=38.2; *P*<.001). Of the 1080 users with high BP at baseline, 590 (54.63%) did not have high BP at their final check. Conversely, of the 5768 users who did not have high BP at baseline, 395 (6.85%) had high BP at their final check (Table 5).

The proportion of smokers was also found to decrease from baseline (1005/8438, 11.91%) to final check (854/8430, 10.13%). This decrease was found to be significant (χ²_1_=48.4; *P*<.001). Of the 1005 smokers at baseline, 308 (30.65%) were not smokers at their final check, whereas 2.11% (157/7436) of the nonsmokers at baseline reported smoking at their final check (Table 5).

**Table 4:** BMI of repeat SiSU users at baseline compared with final check.

Variable	Baseline, mean (SD)	Final check, mean (SD)	Test statistic (Z)	*P* value
BMI (kg/m^2^)	26.37 (7.43)	25.40 (8.06)	−14.24	<.001

**Table 5:** Blood pressure and smoking status of SiSU users at baseline compared with final check

Variables		Baseline, n (%)	Final check, n (%)	Chi-square value (*df*=1)	*P* value
High blood pressure (mm Hg)					
	No	5768 (84.23)	5963 (87.08)	38.2	<.001
	Yes	1080 (15.77)	885 (12.92)	N/A^a^	N/A
**Smoking status**					
	Nonsmoker	7436 (88.09)	7578 (89.87)	48.4	<.001
	Smoker	1005 (11.91)	854 (10.13)	N/A	N/A

^a^N/A: not applicable.

Finally, linear models were used to determine if the number of health checks a user undertakes was significantly related to their final health scores. The baseline scores were controlled in these models.

Due to the skewed distribution of BMI in the final check variables, a generalized linear model with gamma distribution and log link was used to assess the relationship between final BMI measurements and number of health checks. When controlling for baseline measures, the exponentiated coefficient (Exp[b]=0.999; *P*=.23) indicates that BMI at the final check was not significantly associated with the number of health checks (Table 6).

Binary logistic regression demonstrated that an increasing number of health checks were significantly associated with a decreasing likelihood of being a smoker at the final check, although this effect was small (OR 0.959; *P*<.048). There was no significant relationship between the number of health checks and high BP at the final check (OR 0.985; *P*=.14; Table 7).

**Table 6:** Generalized linear model explaining the effect of number of health checks on BMI at final check.

Parameter		Exp (b)^b^ (SE)		*P* value	
Number of health checks		0.999 (1.001)		.23	
BMI at initial check		1.021 (1.000)		<.001	
Gender (reference=female)		1.017 (1.003)		<.001	
**SEIFA^a^ quintile (reference=quintile 5)**				<.001	
	Quintile 1		1.046 (1.005)		
	Quintile 2		1.035 (1.006)		
	Quintile 3		1.033 (1.005)		
	Quintile 4		1.019 (1.004)		
Age		1.001 (1.001)		<.001	

^a^SEIFA: socioeconomic indexes for areas.

^b^Exp(b): exponentiated coefficient.

**Table 7:** Binary logistic regression models explaining the effect of number of health checks on smoking status and high blood pressure status at final check.

Parameter		Smoking status at final check (reference=nonsmoker)				High BP^a^ (mm Hg) at final check (reference=no)			
		OR^b^ (95% CI)		*P* value		OR (95% CI)		*P* value	
Number of health checks		0.959 (0.921-1.000)		.048		0.985 (0.964-1.005)		.14	
High BP at initial check		N/A^c^		N/A		0.113 (0.096-0.132)		<.001	
Smoking status at initial check		0.010 (0.008-0.012)		<.001		N/A^c^		N/A	
Gender (reference=female)		1.074 (0.870-1.324)		.51		1.343 (1.145-1.574)		<.001	
**SEIFA^d^ quintile (reference=quintile 5)**									
	Quintile 1		1.696 (1.277-2.252)		<.001		1.348 (1.075-1.691)		.01
	Quintile 2		1.296 (0.888-1.892)		.18		1.220 (0.907-1.640)		.19
	Quintile 3		1.403 (1.007-1.953)		.045		1.500 (1.183-1.901)		.001
	Quintile 4		1.001 (0.753-1.331)		.99		1.145 (0.919-1.425)		.23
Age		0.991 (0.984-0.998)		.02		1.028 (1.023-1.033)		<.001	

^a^BP: blood pressure.

^b^OR: odds ratio.

^c^N/A: Not applicable.

^d^SEIFA: socioeconomic indexes for areas.

## Discussion

### Principal Findings

At 180,000 health checks, the SiSU wellness dataset is one of the largest datasets ever provided for research, which has been generated by interactive health stations that measure biometric indicators. The scale of this dataset is clearly significant, and this study and the SiSU wellness dataset provide a valuable foundation for extensive investigation into the benefits of health stations in terms of their health monitoring and health promotion capabilities.

This study builds on previous research in the health technology field by providing further insight into the factors that are associated with health kiosk utilization and the potential health benefits of using health kiosks to self-monitor health. Findings indicate that demographics, including gender, age, and SES, were associated with both utilization and repeat utilization of the SiSU health stations, with females, younger people, and those of higher SES using the SiSU health stations at higher rates and more likely to be repeat users. A relationship between health characteristics and repeat utilization of the health stations has also been demonstrated; both higher BMI and high BP at baseline increased the odds of being a repeat user, and smokers were less likely to be repeat users.

The results are consistent with findings regarding the influence of age on the utilization of health technologies, which suggest that younger people use these technologies at higher rates [23-25]. Despite the suggestion by some authors that health kiosks may play a role in reducing this age bias in the use of technology by reducing age-related barriers [29-31], this does not appear to be supported by the results of our study.

Previous studies investigating the influence of gender on health kiosk utilization have demonstrated mixed results. Consistent with our findings, 1 study found that females access health kiosks at higher rates [29], whereas another study found that the relationship between gender and kiosk use is dependent on other demographic variables such as country of birth and SES [32], and other studies have found no relationship between gender and kiosk use [30,33]. The inconsistency of these results is potentially due to differences in the location of the health kiosks (eg, retail environment and hospital setting), which has been found to influence utilization rates [31]; further research could aim to investigate these effects. It is worth noting, however, that although a smaller proportion of SiSU health station users are males, the sheer volume of males undertaking health checks using the SiSU health stations provides a unique opportunity for engaging this demographic, who have been found to access traditional preventative health services at lower rates than females [43,44].

When considering the gender distribution of Priceline Pharmacies’ customer base in conjunction with that of SiSU health stations users, evidence emerges that suggests that the SiSU health stations are highly effective in engaging the male demographic, with 44.13% (79,628/180,442) of SiSU health station users being males. Evidence to support the hypothesis that the SiSU health station is particularly attractive to males in retail environments is further supported by unpublished data from a 6-store retail health station pilot that SiSU wellness undertook with a major Australian Supermarket chain, where males accounted for 54.29% (50,143/92,345) of the checks recorded. In 5 of the 6 supermarkets, males contributed the clear majority of checks, and in the only store where females were the majority, the spread between genders was just 1.40% (Personal Communication by Patrick J Hannebery, May 29, 2019).

The findings regarding the SES of SiSU health station users support previous research that demonstrate that higher SES levels are associated with higher utilization rates of health kiosks [30,31] and health technologies in general [23,25,28]. The poorer rates of utilization in lower SES populations may be the result of a combination of factors, including lower health literacy [45,46]; access barriers such as cost, time, and transport [46,47]; and attitudes toward health care and health care providers [47,48]. Again, location of the health kiosks may play a role in the lower rate of utilization by those from low SES areas.

Our initial descriptive analysis of the health characteristics of SiSU health station users found that SiSU users are generally healthier than the NHS participants on the 4 health characteristics investigated in this study. This supports the findings of previous research, which found that people with chronic disease are less likely to engage in self-monitoring. Such research suggests that poor health is associated with decreased self-efficacy and confidence in health monitoring and improvement, sometimes leading to decreased utilization and adoption of monitoring and prevention [26], which may in part explain these findings. The results of the weighted analysis, however, indicate that age, gender, SES, and place of residence (state) play a large role in the difference in the disease status between SiSU users and the general population, with BP being the only indicator with a substantially large difference between SiSU users and the NHS participants once data were weighted to address the disparity in demographics.

Our investigation of the factors associated with repeat utilization, however, demonstrated that people with high BP are more likely to be repeat users of the health stations and that as BMI increases, the odds of being a repeat user increases. This is an interesting finding in that SiSU users were found to have more favorable outcomes on the 4 health indicators under investigation, whereas repeat users were more likely to have high BP, diabetes, and higher BMIs. These findings provide insight into the differences between once-off utilization of health technologies as opposed to sustained use. The placement of the majority of SiSU health stations in pharmacies may play a role in this relationship, given that those with health issues may be more likely to go to pharmacies on a regular basis.

Regarding repeat utilization of SiSU health stations, our results are somewhat consistent with previous research that demonstrate that people with chronic disease are more likely to use technology to self-monitor their health in a sustained manner [24-27]. For example, higher BMI has been found to be associated with higher frequency of use of mobile health apps [25], whereas those with hypertension have previously demonstrated a higher willingness to consistently self-monitor their health [26]. The difference in the health characteristics of repeat users compared with one-time users is interesting. It has been suggested by some authors that sustained use of self-monitoring may be driven by health-related goals such as weight loss [24,49], which may partially explain these findings.

This study has also provided an initial analysis of the health outcomes of health kiosk users, which to date, have not been established. In doing so, it has paved the way for further investigation into the benefits of such technologies for health monitoring by identifying potential areas for further research.

The findings of our study demonstrate positive initial results in terms of the change in health status between baseline and final health checks for repeat users, although the number of health checks a person undertakes does not appear to influence outcomes at the final check. In terms of the comparison of baseline and final checks, these results are somewhat consistent with much of the research that has been conducted regarding the benefits of various self-monitoring health technologies [6,50,51]; however, they are somewhat inconsistent with the finding that the number of health checks does not impact health outcomes at the final check. For example, previous research has found that the adherent use of digital trackers is associated with weight loss and increased physical activity [6], whereas other studies have found that regular monitoring of BP leads to reductions in BP when combined with other interventions [50,51].

Although the suggestion that self-monitoring is effective when combined with other health interventions is interesting, unfortunately it is difficult from the SiSU data to determine the method through which improvements were achieved (eg, counseling, medication, and physical activity) and, if so, whether the use of SiSU health stations influenced the uptake of these methods. Due to the dearth of research regarding the benefits of technology for self-monitoring, there is also limited information available regarding the possible mechanisms behind the behavior changes that result in the health improvements observed in our study and other studies. Research into the effect of self-monitoring in general on health status suggests that mechanisms may include empowerment or self-efficacy [8,13], self-actualization and self-esteem, as well as greater sensitivity and awareness [6]. With regard to health technologies, there is also some evidence that the aggregation of health data in 1 place provides a more complete picture of health status, enabling more holistic behavior change [14]. Again, these findings present further research opportunities with regard to SiSU health station users.

Finally, there is also some evidence that the benefits of digital health monitoring are more pronounced for people with more serious health problems [6], again demonstrating the potential for further investigation of the SiSU data with regard to this finding.

It is worth noting that the results of all analyses undertaken have the potential to be influenced by the location of the SiSU health stations, that is, pharmacy, retail shopping center, gym, or corporate setting, rural versus metropolitan area, and other location variables. There is potential that the differences in demographics and health status between the SiSU wellness users and NHS participants may be partially explained by the placement of stations across the country, with more health check stations in New South Wales than other states. Repeat utilization and frequency of use of the SiSU health stations are also likely to be influenced by these factors. Future research should aim to investigate the effect of location on the demographic representatives and health characteristics of all users, the factors associated with repeat utilization, and the changes in the health characteristics of repeat users.

Overall, the results of this study are promising in terms of the potential for utilization of SiSU health stations as an effective catalyst for change and a means of empowering consumers to take ownership and achieve improvements in their health status. The considerably large sample size, digitization, and representativeness of health status measures in the data demonstrate the unique value of the SiSU health station data as a cost-effective method of monitoring population health data over time and provides a valuable data source for health workers, population health professionals, policy makers, and researchers alike.

### Limitations

A number of potential limitations of this study have been identified. First, the range of demographic data available in both the SiSU wellness and ABS datasets limit the investigation of the representativeness of the SiSU data. It is possible that there may be underlying differences between the SiSU wellness sample and the general population that cannot be identified through this study. The limited number of demographic variables available for analysis and comparison also mean that the weighting of the SiSU wellness data is restricted to variables available in both the SiSU wellness dataset and the NHS dataset. Furthermore, only a small percentage of the variance in the repeat status of users could be explained by the available variables; additional variables would allow more robust models to be developed to investigate the relationship between demographics, health status, and repeat users more thoroughly.

It must also be acknowledged that the SiSU wellness data and the ABS census/NHS data are not mutually exclusive; in that it is possible that subjects in the SiSU wellness dataset could also be participants in the NHS. Due to this, and as there is no sampling frame to directly compare users and nonusers of the SiSU health stations, interpretation of the results of the comparison of SiSU users and NHS participants should be treated with caution. There are also limitations associated with comparing data from different sources. Although every effort has been made to ensure that the variables from the SiSU health station and NHS data are directly comparable, by collapsing response categories where necessary and using actual measurements to compute categories for variables such as BMI and BP, we were unable to control for potential differences in measurement techniques.

A further limitation is associated with the study design, as it is difficult to determine from the results if the utilization of SiSU health stations has led, directly or indirectly, to the improvements in health characteristics observed in this study. Data available through the SiSU health stations include Prochaska and DiClemente’s *Stages of Change* assessment data, and it is recommended that further studies investigating the relationship between SiSU health station utilization and health outcomes incorporate the relationship between these responses, repeat utilization of health stations, and health outcomes. Additional considerations for further investigation include the presence or absence of other potential drivers of change, including contact with health practitioners and healthy lifestyle programs, and potentially the inclusion of a control group to identify if these improvements are only observed in SiSU users. It must also be noted that the statistical methodology used for this particular research question did not allow for the inclusion of covariates; future research should aim to develop more complex models that involve a wider range of explanatory variables. Finally, the time elapsed between baseline and final check was not considered in these models and could be included in future to identify if changes to health status are more pronounced and/or sustained over time.

For these reasons, although contributing to the small body of research regarding the factors associated with and benefits of health kiosks for self-monitoring health, care should be taken in generalizing these results. Further research should build upon these initial investigations to address some of the identified limitations.

### Conclusions

The findings of this study support much of the previous research regarding the relationship between demographics, health status, and uptake of self-monitoring for health, and, in particular, provide valuable insights regarding the health benefits of health kiosks for the self-monitoring of health. In general, users of SiSU health stations differ from the general population in terms of demographics and are likely to be healthier than the general population. However, this difference in health status appears to be minimal when differences in demographics are taken into account. The results of the study are promising in terms of the potential benefits of using SiSU wellness stations to monitor health; however, there are opportunities for further research into the factors relating to and mechanisms behind these benefits.

## Abbreviations

ABS: Australian Bureau of Statistics
BP: blood pressure
IPW: inverse probability weighting
IRSAD: index of relative advantage and disadvantages
NHS: National Health Survey
OR: odds ratio
SEIFA: socioeconomic indexes for areas
SES: socioeconomic status
